# Predicting histological grade in pediatric glioma using multiparametric radiomics and conventional MRI features

**DOI:** 10.1038/s41598-024-63222-5

**Published:** 2024-06-13

**Authors:** Tengfei Zhou, Baobao Qiao, Bo Peng, Yuqi Liu, Zhenjia Gong, Mengfei Kang, Yu He, Chunying Pang, Yakang Dai, Mao Sheng

**Affiliations:** 1 Department of Radiology, Children’s Hospital of Soochow University, Suzhou, China; 2 School of Life Science and Technology, Changchun University of Science and Technology, Changchun, China; 3 Suzhou Institute of Biomedical Engineering and Technology, Chinese Academy of Sciences, Suzhou, China; 4 Jinan Guoke Medical Engineering Technology Development Co., Ltd, Jinan, China

**Keywords:** Neuroscience, Computational neuroscience

## Abstract

Prediction of glioma is crucial to provide a precise treatment plan to optimize the prognosis of children with glioma. However, studies on the grading of pediatric gliomas using radiomics are limited. Meanwhile, existing methods are mainly based on only radiomics features, ignoring intuitive information about tumor morphology on traditional imaging features. This study aims to utilize multiparametric magnetic resonance imaging (MRI) to identify high-grade and low-grade gliomas in children and establish a classification model based on radiomics features and clinical features. A total of 85 children with gliomas underwent tumor resection, and part of the tumor tissue was examined pathologically. Patients were categorized into high-grade and low-grade groups according to World Health Organization guidelines. Preoperative multiparametric MRI data, including contrast-enhanced T1-weighted imaging, T2-weighted imaging, T2-weighted fluid-attenuated inversion recovery, diffusion-weighted images, and apparent diffusion coefficient sequences, were obtained and labeled by two radiologists. The images were preprocessed, and radiomics features were extracted for each MRI sequence. Feature selection methods were used to select radiomics features, and statistically significant clinical features were identified using t-tests. The selected radiomics features and conventional MRI features were used to train the AutoGluon models. The improved model, based on radiomics features and conventional MRI features, achieved a balanced classification accuracy of 66.59%. The cross-validated areas under the receiver operating characteristic curve for the classifier of AutoGluon frame were 0.8071 on the test dataset. The results indicate that the performance of AutoGluon models can be improved by incorporating conventional MRI features, highlighting the importance of the experience of radiologists in accurately grading pediatric gliomas. This method can help predict the grade of pediatric glioma before pathological examination and assist in determining the appropriate treatment plan, including radiotherapy, chemotherapy, drugs, and gene surgery.

## Introduction

Gliomas are the most common brain tumor among children, with a high incidence rate according to the Central Brain Tumor Registry of the United States^1^. These tumors are classified into two grades: pediatric low-grade tumors (pLGGs) and pediatric high-grade gliomas (pHGGs)^2^. While most children with low-grade gliomas have good overall survival, high-grade gliomas have a poor prognosis and are essentially incurable. Therefore, accurate prediction of glioma is crucial to provide a precise treatment plan to optimize the prognosis of children with glioma^3,4^. Currently, histopathological assessment after surgery or biopsy is the gold standard for glioma grading^5^, but it is challenging to accurately identify the pathological grade from preoperative clinical data.

Gliomas can be visualized on magnetic resonance imaging (MRI). Traditional MRI features capture clinically relevant characteristics observable through conventional radiology assessments by radiologist. For instance, enhancement pattern characterizes the enhancement intensity and pattern post-contrast administration on contrast-enhanced T1-weighted imaging (T1ce), which provides crucial insights into tumor vascularity and enhancement^6,7^. Peritumoral edema can typically be observed on T2-weighted imaging (T2WI) sequences. In T2WI, areas of edema appear as regions of increased signal intensity surrounding the tumor, providing valuable information about the extent and characteristics of the edema surrounding the tumor^8,9^. These imaging features, such as tumor size, location, morphology, enhancement pattern, and peritumoral edema, provide crucial contextual information for pediatric glioma grading. However, traditional MRI features are typically derived from subjective observations and descriptions made by radiologists. While these features offer crucial clinical information, they lack quantitative numerical representation, making them challenging to directly compute and analyze statistically during the analysis process.

Radiomics features extract quantitative characteristics from large amounts of imaging data, including texture features, shape features, and intensity histograms^10,11^, which can provide deeper insights into the histological and biological properties of the tumor^12^. These features can capture subtle variations and irregular patterns in the tumor, enhancing the overall understanding of its characteristics. Recently, radiomics and machine learning techniques have shown great promise in the prediction of glioma grading^13,14^. However, research on the grading of pediatric gliomas using radiomics is limited. Meanwhile, existing methods are mainly based on only radiomics features, ignoring intuitive information about tumor morphology on traditional imaging features.

This study aims is to develop a machine learning model that can incorporate radiomics features and conventional MRI features for improving the prediction of histological grade. The machine learning model will be trained using multiparametric MRI from patients with pediatric glioma. By combining traditional imaging features and radiomics features, we can complement each other’s limitations and improve the accuracy and reliability of grading. Traditional imaging features provide foundational information, while radiomics features offer quantitative details, allowing for a more comprehensive depiction of the tumor's characteristics and enabling more precise grading results.

## Results

### Characteristics of the study cohort

This study analyzed a cohort of 85 patients with gliomas, comprising of 49 males and 36 females aged 0–16 years old. Among them, 28 patients had high-grade gliomas (33%), while 57 patients had low-grade gliomas (67%). No significant differences were found in age and gender between the two groups. The clinical information (gender and age) and tumor characteristics (pathological grades and conventional MRI features) are presented in Table 1.

**Table 1:** The clinical information and tumor characteristic of the whole cohort.

Variables	Total cases	pLGGs	pHGGs	*p*
Subject Number	85	57	28	–
Gender (M/F)	49/36	31/26	18/10	0.3850
Age (Mean ± SD)	5.80 ± 3.39	6.11 ± 3.49	5.18 ± 3.19	0.2400
Histological grades				
Grade I (n = 36)	36	36	–	–
Grade II (n = 21)	21	21	–	–
Grade III (n = 21)	21	–	21	–
Grade IV (n = 7)	7	–	7	–
Histopathology classification				
Pilocytic astrocytoma	20	20	0	–
Diffuse astrocytoma	27	26	1	–
Dysembryoplastic neuroepithelial tumor	3	3	0	–
Ganglioglioma	3	3	0	–
Oligodendroglioma	4	3	1	–
Ependymoma	22	2	20	–
Diffuse midline glioma and high-grade glioma	6	0	6	–
Conventional MRI features				
Location (supratentorial/infratentorial)	35/50	26/31	9/19	0.2406
Cystic grade (solid/cystic)	34/51	28/29	6/22	0.2804
Diffusion restriction (Yes/No)	38/47	18/39	20/8	< 0.001
Edema grade (Yes/No)	53/32	29/28	24/4	0.2477
Cross the midline (Yes/No)	50/35	33/24	17/11	0.8068
Ring enhancement (Yes/No)	37/48	31/26	6/22	0.0307
Hydrocephalus (Yes/No)	43/42	26/31	17/11	0.1950

### Selected radiomics features

**Figure 1:**
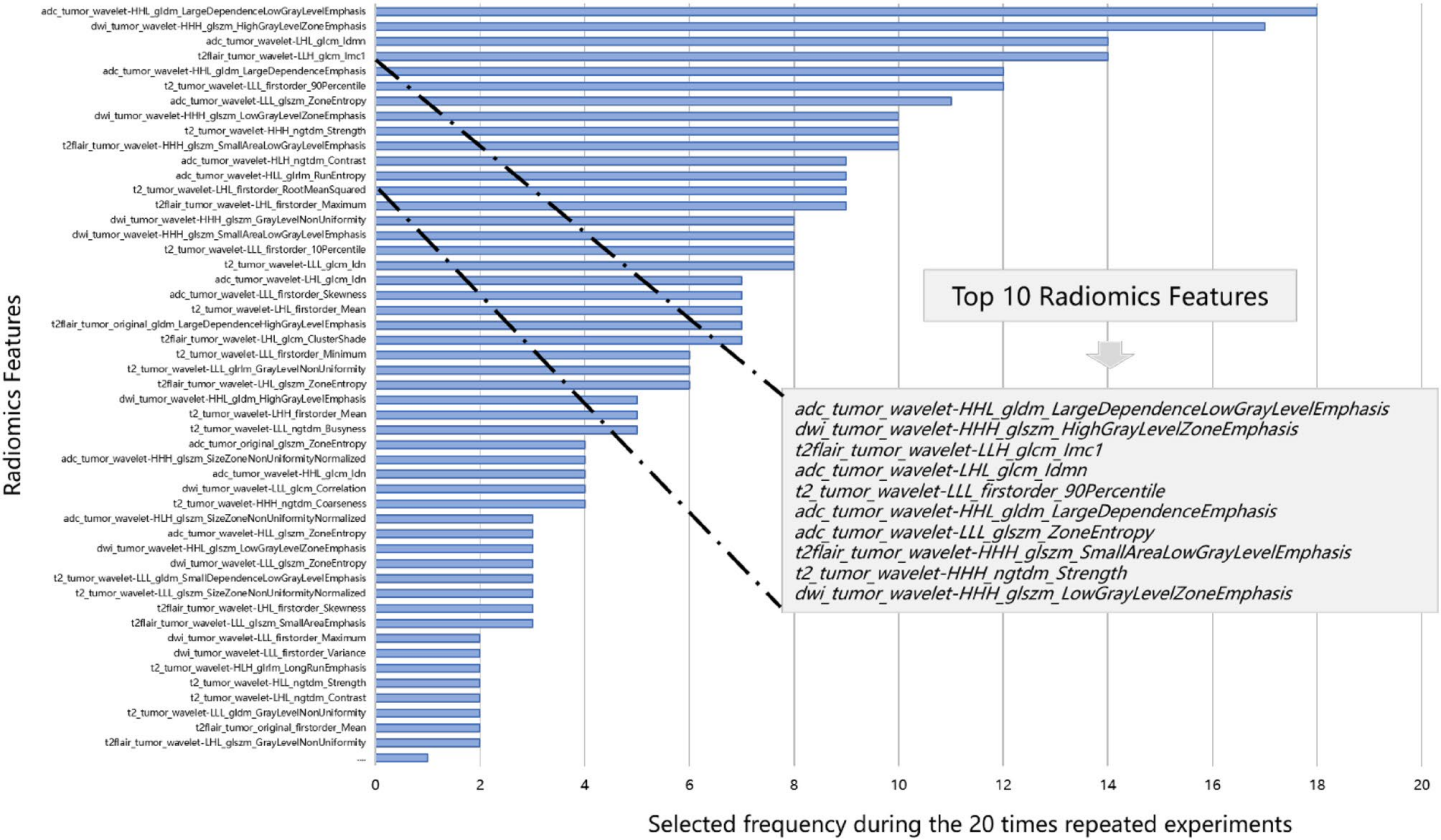
The importance ranking of radiomics features and the TOP 10 radiomics features.

Radiomics features extracted from each MRI sequence were helpful in grading pediatric gliomas. The importance ranking of radiomics features is shown in Fig. 1, with the TOP 10 radiomics features listed below:

t2flair_tumor_wavelet_HHH_glszm_SmallAreaLowGrayLevelEmphasis;

adc_tumor_wavelet_HHL_gldm_LargeDependenceEmphasis;

adc_tumor_wavelet_LLL_glszm_ZoneEntropy;

cet1_tumor_wavelet_LHL_glszm_SmallAreaEmphasis;

cet1_tumor_wavelet_LHH_glszm_LowGrayLevelZoneEmphasis;

adc_tumor_wavelet_LHL_glcm_Idmn;

dwi_tumor_wavelet_HHH_glszm_HighGrayLevelZoneEmphasis;

t2_tumor_original_firstorder_Range;

t2flair_tumor_wavelet_LLH_glcm_Imc1;

dwi_tumor_wavelet_HHH_glszm_SmallAreaLowGrayLevelEmphasis.

These TOP 10 radiomics features included three ADC (apparent diffusion coefficient ) wavelet features, two CET1WI (contrast-enhanced T1-weighted imaging) wavelet features, two T2 FLAIR (T2-weighted fluid-attenuated inversion recovery) wavelet features, two from diffusion-weighted images (DWI) wavelet features, and one T2 original feature.

### Selected conventional MRI features

**Figure 2:**
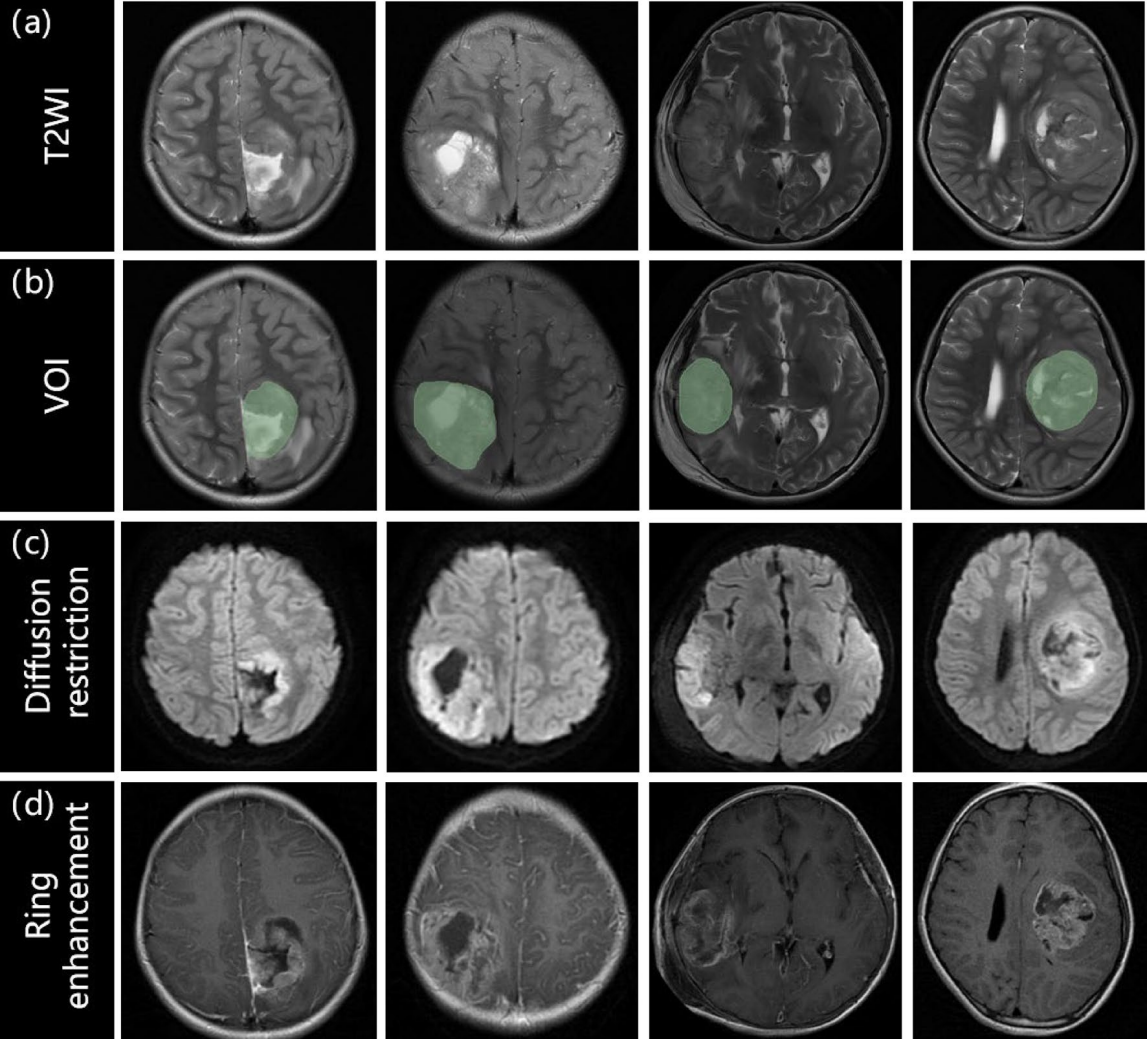
The visualization of original T2WI images, VOI, diffusion restriction, and ring enhancement for different patients with glioma. The histological grades from left to right is grade III, III, IV, and IV, respectively. pHGGs with histological grade of III–IV typically exhibit characteristics of restricted diffusion and ring-enhancement on imaging. For instance, due to the large size of tumor cell nuclei and dense cell arrangement, the intercellular and extracellular spaces are narrow, resulting in restricted diffusion of water molecules. Moreover, the high metabolic rate of tumor cells and the tight accumulation of cells around the necrotic area promote the aggregation of neovascularization, typically appearing as thick-walled ring enhancement in the enhanced images of high-grade tumors. These two conventional MRI features can help distinguish high-grade tumors.

The conventional MRI features were selected using *t*-tests, and significant differences were observed between pLGGs and pHGGs groups for diffusion restriction and ring enhancement (diffusion restriction: *p* < 0.001; ring enhancement: *p* = 0.0307). The detailed statistical results of tumor characteristics are presented in Table 1. Diffusion restriction was mainly observed as hyperintensity at the tumor on DWI images, as demonstrated in Fig. 2c. Whereas ring enhancement was mainly observed as one or more ring-shaped enhancement foci of the tumor on CET1WI image as illustrated in Fig. 2d.

The features types used to train the classifier model were shown in Table 2. The feature set ${\text{F}}_{0}=[{R}_{1},\ldots ,{R}_{n}]$ only contained radiomics feature *R* of the five image modalities. The feature set ${\text{F}}_{1}=[{R}_{1},\ldots ,{R}_{n}, {C}_{1}]$ contained radiomics feature *R* and clinical factor of diffusion restriction. The feature set ${\text{F}}_{2}=[{R}_{1},\ldots ,{R}_{n}, {C}_{2}]$ contained radiomics feature *R* and clinical factor of ring enhancement. The feature set ${\text{F}}_{3}=[{R}_{1},\ldots ,{R}_{n}, {C}_{1},{C}_{2}]$ contained radiomics feature *R* and the two clinical factors.

**Table 2:** Details of the radiomics features and conventional MRI features used in training of the AutoGluon classifier.

Feature types	Explanation
${\text{F}}_{0}=[{R}_{1},\ldots ,{R}_{n}]$: Radiomics features	*n* radiomics features for CETWI, T2WI, T2 FLAIR, DWI, ADC (*n* = 4255)
${\text{F}}_{1}=[{R}_{1},\ldots ,{R}_{n}, {C}_{1}]$: Radiomics features + diffusion restriction	
${\text{F}}_{2}=[{R}_{1},\ldots ,{R}_{n}, {C}_{2}]$: Radiomics features + ring enhancement	${C}_{1}$: diffusion restriction (*Yes* = 1, *No* = 0)
${\text{F}}_{3}=[{R}_{1},\ldots ,{R}_{n}, {C}_{1},{C}_{2}]$: Radiomics features + diffusion restriction + ring enhancement	${C}_{2}$: ring enhancement (*Yes* = 1, *No* = 0)

### Performance of the classification model

To evaluate the effectiveness of radiomics features in grading pediatric gliomas, we employed AutoGluon-based model and SVM-based model (such as C-SVC and Nu-SVC). Table 3 shows the classification performance of C-SVC, Nu-SVC and AutoGluon using radiomics features. Compared to the traditional SVM-based classifier model, the AutoGluon model achieves better performance. The AUC of glioma grading using the AutoGluon model is 0.8071, while the classification accuracy of the two SVM-based classifiers is 0.7742 (C-SVC) and 0.7484 (Nu-SVC), respectively. It can be seen that the classification accuracy of the AutoGluon model is improved by 0.0329–0.0587.

**Table 3:** Classification performance of classifier of C-SVC, Nu-SVC and AutoGluon frame using different features.

Machine learning model	AUC	BACC	SEN	Precision	W_F1	*p* value
C-SVC-R	0.7742	0.7355	0.7166	0.6586	0.6587	< 0.0001
Nu-SVC-R	0.7484	0.6053	0.3833	0.7041	0.6041	< 0.0001
AutoGluon-R	0.8057	0.6609	0.3809	0.7256	0.6967	0.7514
AutoGluon-C	0.6816	0.6177	0.3681	0.3750	0.6355	0.0170
AutoGluon-RC	0.8071	0.6659	0.3917	0.7565	0.7014	–

Feature type: -R = Radiomic features, -C = Conventional MRI features, -RC = Radiomic features + Conventional MRI features.

**Figure 3:**
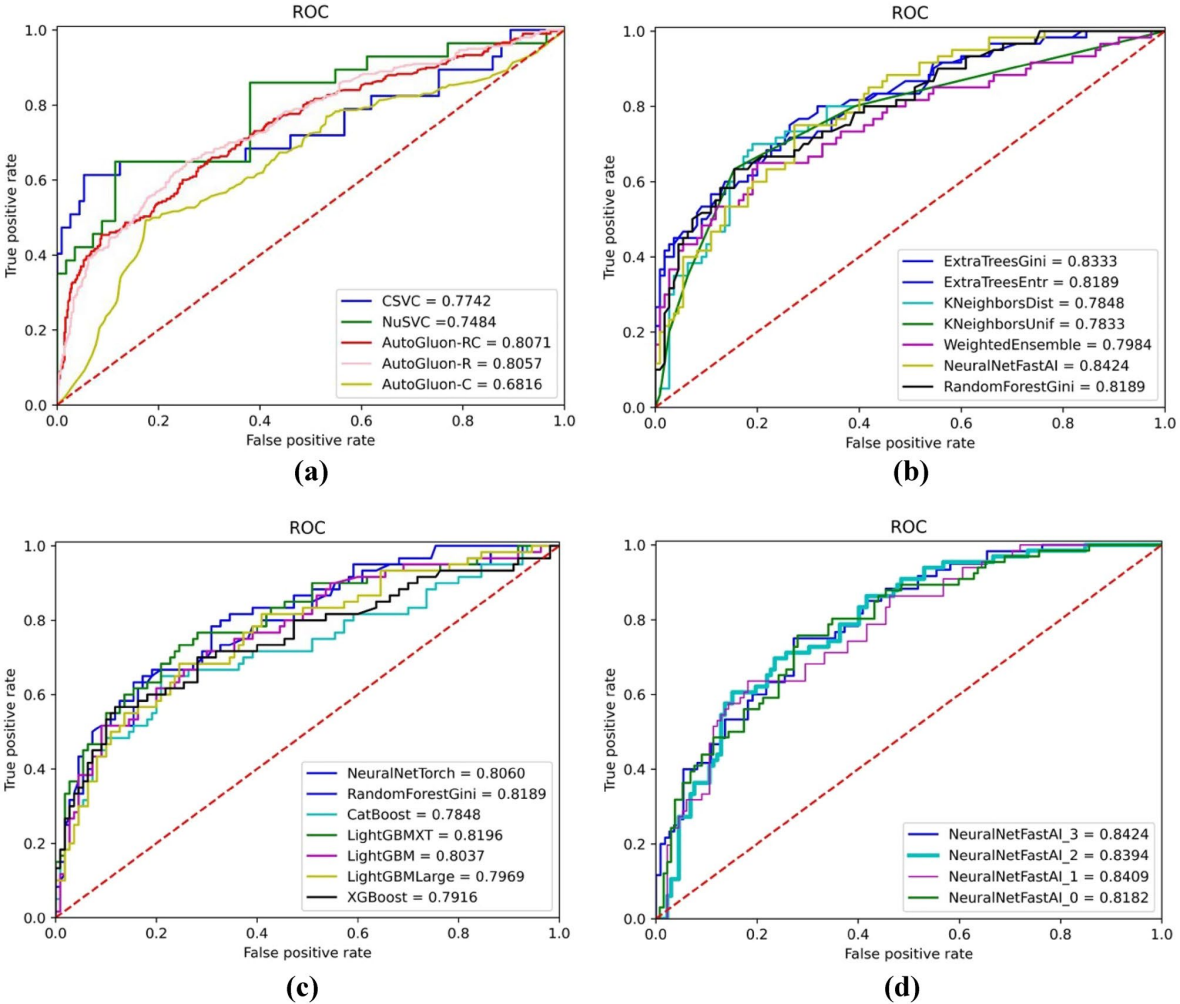
The receiver operating characteristic (ROC) curves. (**a**) The ROC curves of C-SVC, Nu-SVC and AutoGluon framework, (**b**,**c**) The ROC curves of the classifier models under the AutoGluon framework, and (**d**) The ROC curves of the NeuralNetFastAI model using different feature types.

**Figure 4:**
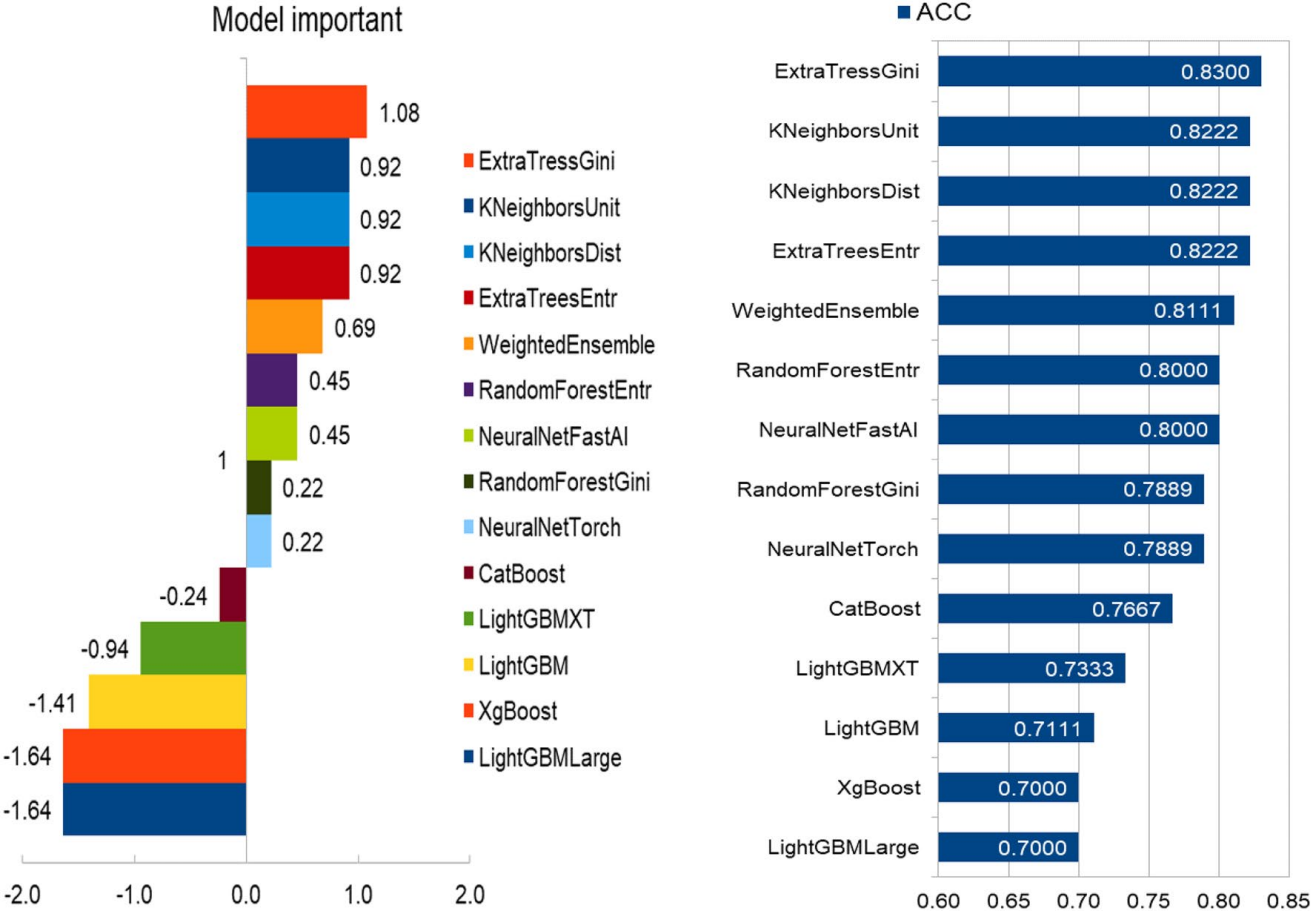
Importance of multimodal variables and performance of base machine learning models predicting histological grade of pediatric Glioma. The model importance is generated by the formula: importance = (xi − x)/σ, where xi is the ACC of each model dedicated, and x and σ are the mean and standard deviation of all ACCs, respectively.

To further investigate the performance of different classification models under the AutoGluon framework, we separately calculated the classification performance of the base thirteen classifier models using only radiomics features. According to the classification accuracy, the outstanding performed classifier models are NeuralNetFastAI, ExtraTreesGini, LightGBMXT, ExtraTreesEntr, and RandomForestGini, with classification accuracy of 0.8424, 0.8333, 0.8196, 0.8189, and 0.8189, respectively. Table 4 displays the classification performance of the five classifier models under the AutoGluon framework using radiomics features for predicting histological grade. Figure 3a shows the receiver operating characteristic (ROC) curves of the five classifier models that performs best under the AutoGluon framework. Different sets of features are used to train machine learning models. AutoGluon-R only uses Radiomic features, AutoGluon-C only uses Conventional MRI features, while AutoGluon-RC uses both Radiomic features and Conventional MRI features. The experimental results show that among the classifiers used, AutoGluon-RC performed the best with an AUC value of 0.8071, slightly higher than AutoGluon-R's 0.8057. The AUC values for CSVC and NuSVC are 0.7742 and 0.7484, respectively, slightly lower than those of the AutoGluon series. AutoGluon-C achieved the lowest AUC value of 0.6816. These results indicate that AutoGluon-RC has demonstrated the most superior performance in this classification task. The experimental results using the AutoGluon classification framework with different base classifiers for pediatric glioma grading are shown in Fig. 3b and c. These results demonstrate that the classifiers with the highest AUC values are NeuralNetFastAl (AUC = 0.8424), followed by ExtraTreesGini (AUC = 0.8333) and ExtraTreesEntr (AUC = 0.8189). Figure 4 displays the model importance and performance of base classifiers in AutoGluon framework. These results demonstrate that the NeuralNetFastAI classifier model achieved the best performance in pediatric glioma grading.

**Table 4:** Classification performance of the best five classifier models in AutoGluon using radiomic features and two C features to predict histological grade of pediatric Glioma.

Classifier model	AUC	BACC	SEN	Precision	W_F1
CatBoost	0.7848	0.6916	0.4833	0.7026	0.7230
ExtraTreesEntr	0.8189	0.6704	0.3500	0.8833	0.7127
ExtraTreesGini	0.8333	0.6863	0.4000	0.8416	0.7257
KNeighborsDist	0.7848	0.6431	0.3500	0.7654	0.6837
KNeighborsUnif	0.7833	0.6431	0.3500	0.7654	0.6837
LightGBM	0.8037	0.6598	0.3833	0.7416	0.6966
LightGBMLarge	0.7969	0.6068	0.2500	0.6371	0.6387
LightGBMXT	0.8196	0.6325	0.2833	0.8416	0.6780
NeuralNetFastAI	0.8424	0.6780	0.4833	0.5965	0.6994
NeuralNetTorch	0.8060	0.6196	0.2666	0.7333	0.6541
RandomForestEntr	0.8174	0.6765	0.4166	0.7683	0.7145
RandomForestGini	0.8189	0.7053	0.4833	0.7750	0.7408
WeightedEnsemble_L2	0.7984	0.6856	0.4166	0.7916	0.7245
XGBoost	0.7916	0.7242	0.5667	0.7483	0.7441

Additionally, conventional MRI features were combined with radiomics features to improve the classification performance. Table 5 illustrates the classification performance of different feature types using ExtraTressGini model. Figure 3d shows the ROC curves of NeuralNetFastAI model using different feature types. For feature type ${\text{F}}_{3}$ (radiomics features + diffusion restriction + ring enhancement), NeuralNetFastAl achieves the highest AUC of 0.8424, indicating the best performance. For feature type ${\text{F}}_{2}$ (Radiomics features + ring enhancement), NeuralNetFastAl achieves an AUC of 0.8394, showing slightly lower performance compared to type 3. For feature type ${\text{F}}_{1}$ (Radiomics features + diffusion restriction), NeuralNetFastAl achieves an AUC of 0.8409, performing similarly to type ${\text{F}}_{2}$. For feature type ${\text{F}}_{0}$ (only radiomics features), NeuralNetFastAl achieves the lowest AUC of 0.8182. The experimental results indicate the varying impact of different feature types on the performance of the NeuralNetFastAl classifier. These results show that the performance of pediatric glioma grading using AutoGluon is improved by combining radiomics features and conventional MRI features.

**Table 5:** Classification performance of different feature types under the NeuralNetFastAI model.

Classifier model	Feature types	AUC	BACC	SEN	Precision	W_F1
NeuralNetFastAI	${\text{F}}_{0}$	0.8182	0.6773	0.5000	0.7100	0.7153
NeuralNetFastAI	${\text{F}}_{1}$	0.8409	0.7227	0.6000	0.7547	0.7482
NeuralNetFastAI	${\text{F}}_{2}$	0.8394	0.7091	0.6000	0.6719	0.7324
NeuralNetFastAI	${\text{F}}_{3}$	0.8424	0.6780	0.4833	0.5965	0.6994

## Discussion

Pediatric gliomas not only differ significantly from adult gliomas in terms of incidence, site of occurrence, and prognosis but also exhibit obvious differences in terms of pathogenesis and molecular characteristics. Therefore, the grading model for adult gliomas cannot be simply applied to children. Developing a preoperative grading classification model for pediatric gliomas is of great value for improving the prognosis of affected children.

In this study, we developed and validated a classification model that enables the integration of radiomics features and conventional MRI features to improve the predication of histological grades in pediatric glioma. First, our AutoGluon grading model for pediatric glioma grading was superior to SVM-based classification models. Second, the expertise of experienced radiologist plays an important role in the grading of pediatric gliomas. The experiment results confirm this point that incorporating conventional MRI features with radiomics features can help improve the classification accuracy of machine learning model. Finally, our results show promise of our AutoGluon grading model and potential utilities for pediatric glioma histological grades predicting.

Radiomics features offer crucial information for glioma grading. These features comprise traditional image features such as first-order gradient features, shape features, texture features, as well as various features after geometric filtering and transformation. After feature selection, thirty radiomics features were reserved and ranked based on their frequency of occurrence using in classification experiment, as shown in Fig. 1. The TOP 10 radiomics features included all five modality (CET1WI, T2WI, T2 FLAIR, DWI, and ADC sequences) images, indicating that each modality contributes to pediatric glioma grading. And nine of the TOP 10 radiomics features were wavelet features, suggesting that wavelet texture features can provide multiple frequencies and scales information about tumor heterogeneity using wavelet decomposition.

While the development of radiomics technology has indeed influenced the grading methods for pediatric gliomas, traditional MRI features still hold significance in the grading process. Firstly, traditional MRI features provide essential information regarding the extent of tumor infiltration, compression of adjacent structures, and presence of necrosis, all of which contribute to the overall assessment of tumor aggressiveness and prognosis^8,9^. Additionally, traditional MRI features are readily identifiable and interpretable by radiologists, facilitating quick and accurate diagnosis in clinical practice. Therefore, traditional MRI features remain relevant and complementary in the assessment of pediatric gliomas, providing valuable insights into tumor characteristics and behavior.

In this study, significant differences between pHGGs and pLGGs can be observed on MRI images for restricted diffusion and ring enhancement. Restricted diffusion can provide information of the diffusion of microscopic water molecules in normal and diseased tissues^15,16^. In malignant tumors, due to the large nuclei of tumor cells, increased nuclear-to-cytoplasmic ratio, and tight cell arrangement, the intracellular and extracellular spaces are narrowed, and the diffusion of water molecules is restricted. Whereas in benign tumors, due to the characteristics of low cell density and loose cytoplasm, the diffusion of water molecules is generally not restricted, and tumor lesions show low signal on DWI^13^. In this study, the classification performance has been improved by using diffusion restriction combined with radiomics characteristics in pediatric gliomas grading, indicating that limited diffusion can be used as an important clinical indicator for differentiating the grading of childhood gliomas.

The high metabolic rate and tight cell packing of tumor cells promote the accumulation of new blood vessels around necrotic areas, which are usually thick-walled ring enhancements on enhanced images^14^. This enhancement pattern can be seen in high-grade astrocytoma and anaplastic ependymoma. Wu et al. combined ring enhancement with radiomics features to predict H3K27M mutation in childhood high-grade gliomas, and concluded that ring enhancement can be used as a clinical predictor of H3K27M mutation^17^. Meanwhile, Chiang et al. and Hoffman et al. found that ring enhancement was an important factor in poor overall survival in patients with pontine glioma^18,19^. In conclusion, ring enhancement can also be a predictor of molecular mutation and prognosis of glioma. However, studies using ring enhancement as a predictor of the pediatric glioma grading are rare. This study shows that the combination of clinical factors and radiomic features can better predict the pediatric gliomas grades than using radiomic features alone.

**Figure 5:**
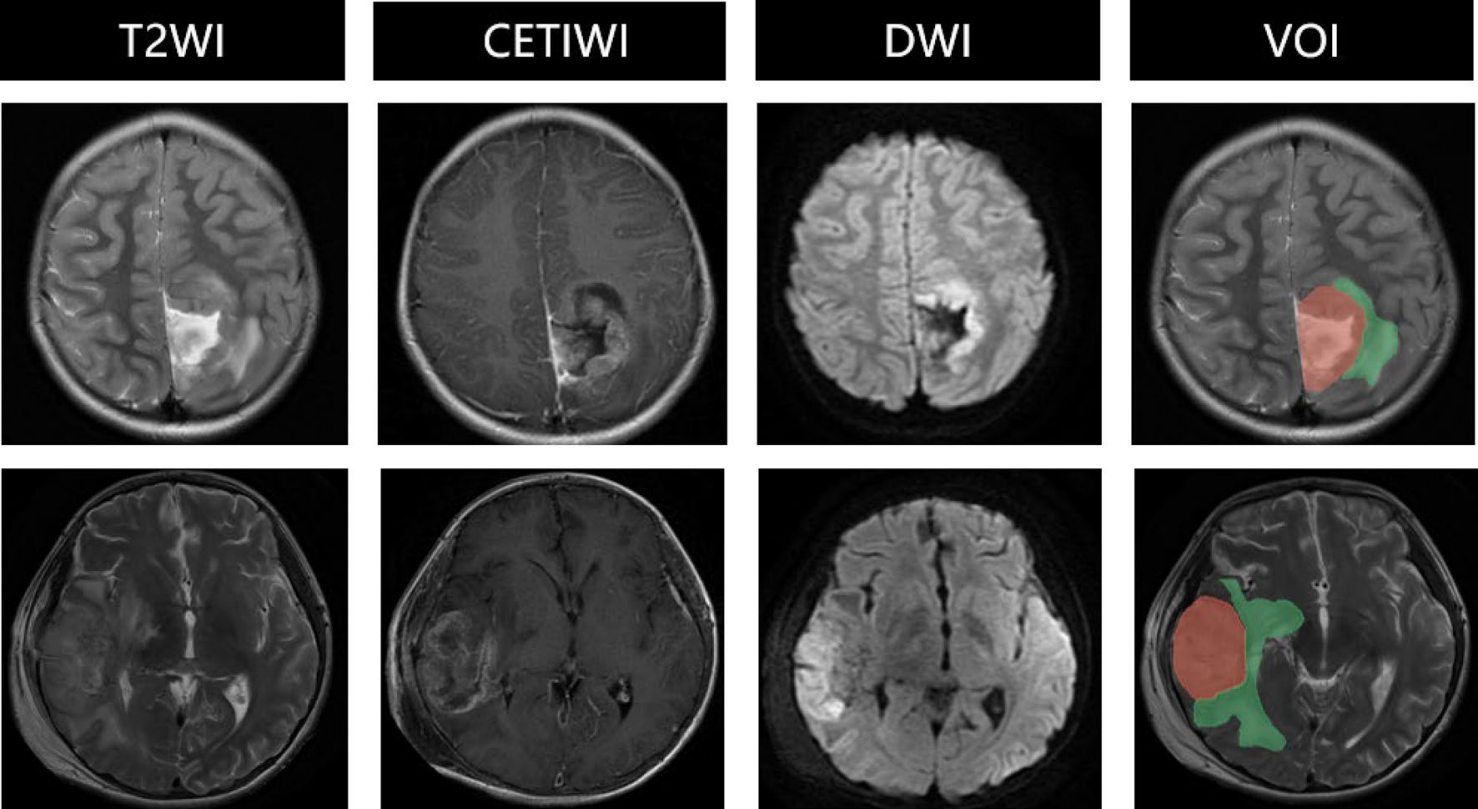
Visualization of the differences in tumor region definition between this study and BRATS public dataset. The two rows represent two subjects. In the VOI (Volume of Interest) images, the red region represents the tumor region defined in this study, while the green region represents the peritumoral edema. The labels in BRATS include the peritumoral edema, whereas in this study, the tumor region mainly refers to the intratumoral necrotic, cystic degeneration, and hemorrhagic.

There are several limitations in this study. First, more data from several centers needs continually collecting. Second, although the World Health Organization includes molecular type information for glioma grading in 2021. This study data was collecting from 2015 to 2022, and only part of participants had molecular type information, so the classification of pHGGs and pLGGs was based on histological grade. In fact, molecular type and histological grade are both essential for analysis of glioma in clinical situation. Third, although radiomics are useful for pediatric gliomas grading, the tumors exhibit high heterogeneity as pilocytic astrocytoma. Further research is needed to explore the relationship between radiomics features and tumor heterogeneity by using advanced MRI sequences, including insights into white matter fiber orientation and integrity (DTI), tumor hemodynamics (DSCE), and tumor metabolism (MRS). By integrating these data, it becomes possible to get information about the orientation and integrity of white matter fibers, the hemodynamic characteristics of tumors, and the information about tumor metabolism^20^. Fourth, the tumor region defined in this study includes cystic components, necrosis, and hemorrhage, which corresponds to the “TC” region (the solid part of the tumor) defined in the BRATS public dataset (shown in Fig. 5). In following research, we will investigate the association between the tumor edema region and the pediatric gliomas grades.

In conclusion, we developed an AutoGluon model using multiparametric radiomics features and conventional MRI features to predict the histological grade of pediatric gliomas in this study. The model demonstrated outstanding classification performance in distinguishing between pHGGs and pLGGs. The results show that incorporating conventional MRI features can help improve the classification performance, indicating the crucial role of clinical factors in accurately assessing glioma grade. This method can assist clinicians in predicting the histological grade of gliomas prior to surgery, making treatment decisions of radiotherapy, chemotherapy, targeted drugs, and genetic surgery.

## Methods

### Participants

We collected clinical information and MRI data from 91 patients diagnosed with grade I–IV gliomas according to the World Health Organization (WHO) Classification of Tumors of the Central Nervous System^2^, from 2015 to 2022. Patients with WHO grade I and grade II gliomas were classified as pediatric low-grade glioma (pLGGs), while patients with WHO grade III and grade IV gliomas were classified as pediatric high-grade glioma (pHGGs). This study was approved by the Ethics Committee of the Children's Hospital of Soochow University. And written informed consent was obtained from all participants. The study was conducted according to the latest version of the Declaration of Helsinki.

**Figure 6:**
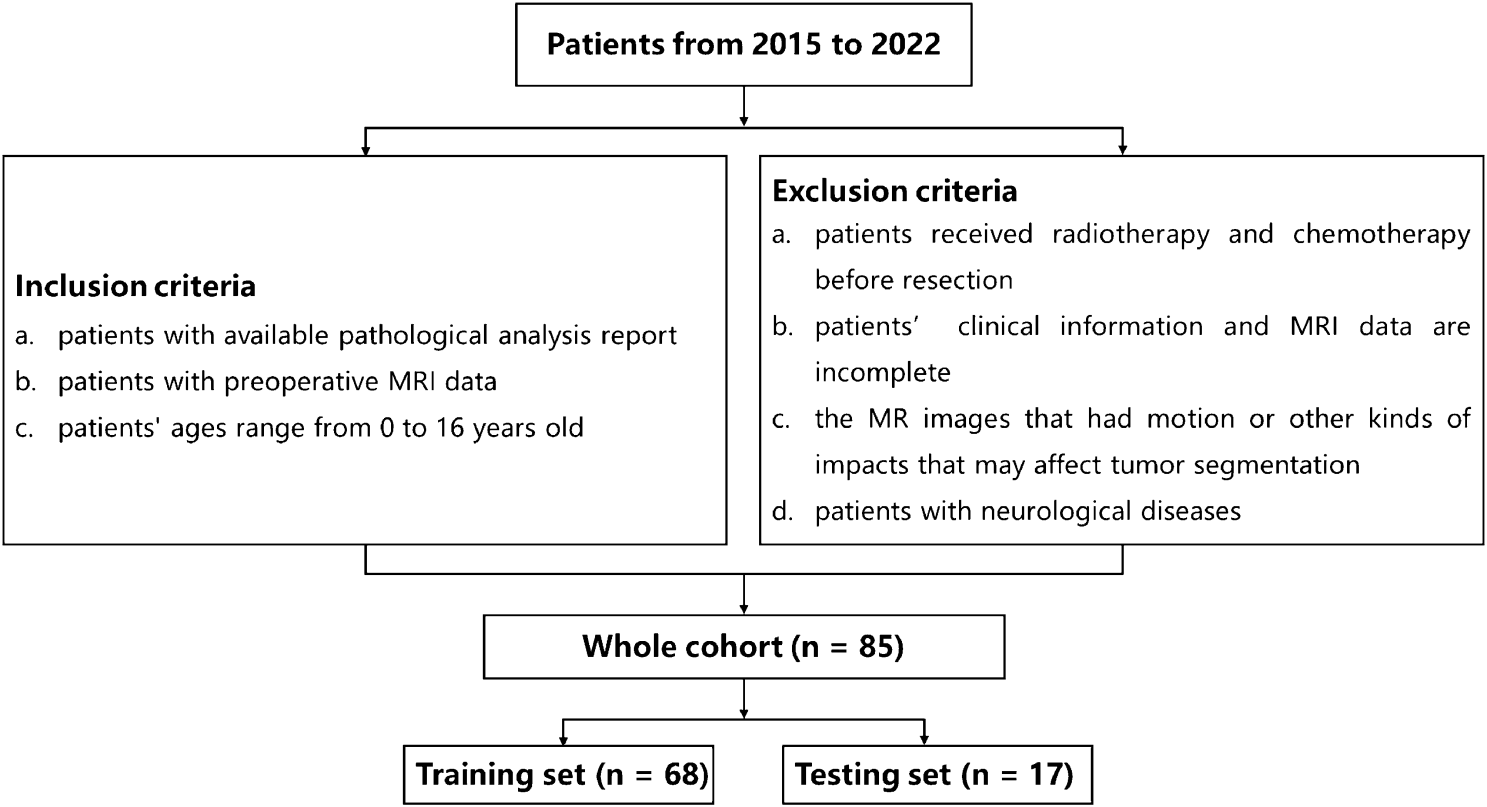
The inclusion and exclusion criteria for collection of the patients and the data splitting.

The study inclusion criteria were as follows: (1) patients with available pathological analysis report; (2) patients with preoperative MRI data; (3) patients aged between 0 and 16 years old. Six patients were excluded due to the following conditions: (1) patients who received radiotherapy and chemotherapy before resection; (2) patients whose clinical information and MRI data were incomplete; (3) MR images that had motion or other kinds of artifacts that may affect tumor segmentation; (4) patients with neurological diseases. Finally, 85 patients met the study requirements (57 pLGGs and 28 pHGGs). The inclusion and exclusion criteria for patient selection and the data splitting process are illustrated in Fig. 6.

### MRI acquisition

The multiparametric MRI images were acquired on a 3 T MRI system (GE Discovery MR750W) with an 8-channel phased-array head coil, including CET1WI, T2WI, T2 FLAIR, DWI, and ADC. The ADC sequence is derived from the DWI sequence, providing quantitative measurements of diffusion within tissues. While the DWI sequence displays the movement of water molecules within tissues, the ADC sequence offers quantitative metrics of this diffusion. Therefore, the combined use of these two sequences provides comprehensive and accurate information, aiding in the imaging diagnosis and assessment of gliomas. The CET1WI, T2WI, T2 FLAIR, DWI and ADC were obtained with repetition time/echo time (TR/TE) = 1750/25 ms, repetition time/echo time (TR/TE) = 5800/100 ms, repetition time/echo time (TR/TE) = 9800/90 ms, repetition time/echo time (TR/TE) = 5000/80 ms, repetition time/echo time (TR/TE) = 5000/80 ms. All MRI sequences were obtained with filed of view (FOV) = 220 × 220 mm, slice thickness = 5.0 mm, slice spacing = 1.0 mm, matrix = 256 × 256. B value of DWI were 1000 s/mm^2^ and 0 s/mm^2^. During scanning, most of the patients were able to remain in natural sleep. Patients who were unable to cooperate were sedated with chloral hydrate (0.5 g in 10 ml, 0.5 ml/kg body weight) 30 min prior to scanning.

### Data preprocessing

A volume of interest (VOI) was manually segmented on T2WI slice-by-slice by two neuroradiologists with 5 years of experience each and verified by a senior neuroradiologist with over 25 years of experience. All three radiologists were blinded to the clinical and pathological reports. The VOI was segmented using ITK-SNAP tool (http://www.itksnap.org/). Because the occurrence of intratumoral necrotic, cystic degeneration, and hemorrhagic usually indicates a higher heterogeneity of the tumor, which is helpful for histological grading, whereas the association between the peritumoral edema and the histological grade is relatively weak^21^. The VOI was defined as the entire tumor volume, including intratumoral necrotic, cystic degeneration, and hemorrhagic, whereas peritumoral edema was excluded.

The data preprocessing steps included: (1) CET1WI, T2 FLAIR, DWI, and ADC images were co-registered to T2WI for each case using the Elastix toolbox; (2) resampling of all images into a uniform voxel size of 1 × 1 × 1 mm^3^; (3) implementation of N3 bias correction to correct MRI bias field; and (4) standardization of image intensities to a range of 0–255 to reduce the influence of intensity inconsistency.

### Radiomics feature extraction and selection

The radiomics features were extracted using PyRadiomics on Python 3.9. 851 radiomics features were extracted from each VOI, including 18 first-order gray-level statistic features, 14 3D shape-based features, 24 Gy-level co-occurrence matrix (GLCM) features, 16 Gy-level run length matrix (GLRLM) features, 16 Gy-level size zone matrix (GLSZM) features, 5 neighboring gray tone difference matrix (NGTDM) features, and 14 Gy-level dependence matrix (GLDM) features, and 744 wavelet features. The online documentation contains a detailed description of the radiomics features (https://pyradiomics.readthedocs.io/en/latest/features. html). A total of 4255 radiomics features were extracted for each subject across the five sequences. The feature values were normalized to [0, 1] using min–max normalization.

Feature selection was conducted using* t*-test and Max-Relevance and Min-Redundancy (mRMR). First, a statistical *t*-test was performed on the features, retaining only those that exhibited statistical significance (*p* < 0.05) between pLGGs and pHGGs. Second, the mRMR technique was employed to select features based on their mutual information and statistical dependence, which identified features with the highest correlation and lowest redundancy between pLGGs and pHGGs.

### Conventional MRI features extraction and selection

The conventional MRI features were evaluated by two radiologists who were blinded to the clinical pathological grades, based on multiparametric MRI images. The visualization of diffusion restriction and ring enhancement for different grades of gliomas were depicted in Fig. 2.The assessment criteria for conventional MRI features were as follows: (1) Location: tumors were classified as Supratentorial or Infratentorial (supratentorial = 1, infratentorial = 0) based on the tentorium; (2) Cystic grade: tumors with cystic components greater than 50% were classified as mainly cystic, while those with cystic components less than 50% were considered mainly solid (solid = 1, cystic = 0); (3) Diffusion restriction: tumors that appeared hyperintense on DWI images and showed a corresponding decrease in the apparent diffusion coefficient (ADC) value were considered positive (Yes = 1, No = 0); (4) Peritumoral edema: regions with no enhancement and hyperintensity outside the tumor parenchyma on T2 FLAIR images were classified as positive (Yes = 1, No = 0); (5) Cross the midline: tumors that extended diffusely across the midline of the brain were classified as positive (Yes = 1, No = 0); (6) Ring enhancement: tumors with one or more ring-shaped enhancement foci on CET1WI images, with the center showing no enhancement and appearing hypointense, were classified as positive (Yes = 1, No = 0); (7) Hydrocephalus: distension of the brain ventricular system with periventricular hyperintense interstitial edema on T2 FLAIR images were classified as positive (Yes = 1, No = 0). The assessment results of the conventional MRI features were compiled into a feature vector for each subject.

To determine if there were significant differences in conventional MRI features between pHGGs and pLGGs groups, a *t*-test was performed. The *p* value was calculated and presented in Table 1. The conventional MRI features that were found to be statistically significant were diffusion restriction and ring enhancement, which were included in training the classifier alongside the selected radiomics features.

### Classifier modeling

**Figure 7:**
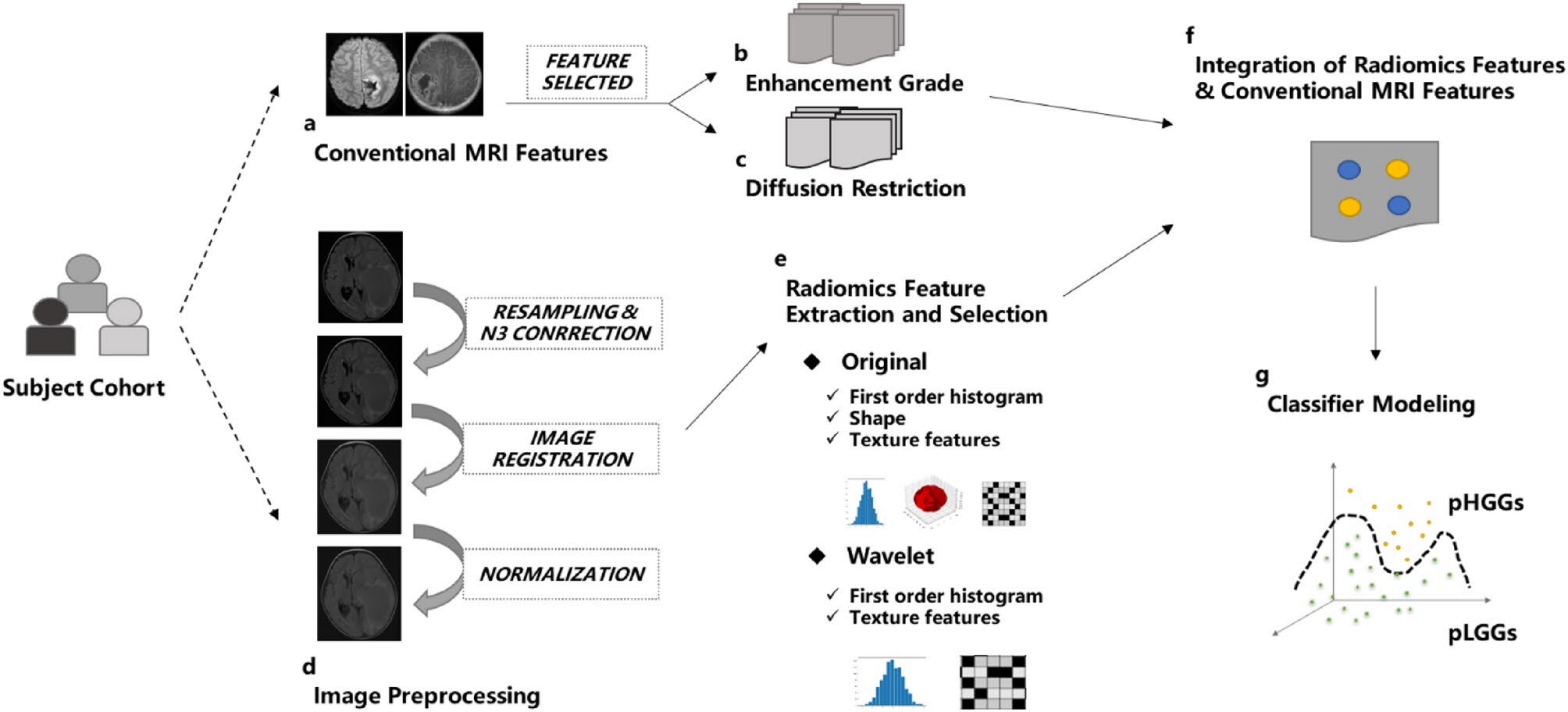
The entire flowchart of classifier modeling using radiomics features and conventional MRI features for prediction pHGGs and pLGGs.

The classifier model was based on the open-source AutoGluon platform, which is a state-of-the-art AutoML framework that utilizes multiple models and employs a novel form of multi-layer stack ensembling. The first layer of the model consists of various base models, including extreme random trees, k-nearest neighbors, gradient boosting machines, random forests, and tabulated neural networks. The outputs of these learners are concatenated and fed into the next layer of the model, which comprises multiple stacker models. These stackers serve as the base models for additional layers. AutoGluon employs random search for hyperparameter tuning, model selection, ensembling, feature selection, data pre-processing, and data splitting. The entire flowchart of the classifier modeling process is presented in Fig. 7.

### Training and testing

All subjects were randomly divided into 80% and 20% for training and testing. The training samples with the selected features were used to train the AutoGluon model. A fivefold cross-validation was used for hyperparameter tuning. The testing samples were fed into the fine-tuned training model. To reduce the variance of the classification results due to the small sample size, the dividing of train-test splits was repeated 10 times. The number of LGGs and HGGs in each of the 10 train-test split is shown in Table 6. The positive and negative sample distribution in the training and testing sets followed a similar ratio as that of the entire dataset. This data splitting approach determined the number of positive and negative samples in the training and testing sets based on the proportion of positive and negative samples in the entire dataset. By maintaining a similar proportion of positive and negative samples in both the training and testing sets, it ensured that the model faces a realistic class distribution during training and testing. The model performance was evaluated using the average observations of the repeated experiments.

**Table 6:** The number of LGGs and HGGs in each of the 10 train-test splits.

Category	Training	Testing
Total	80% of total subjects	20% of total subjects
LGGs	80% of total LGGs	20% of total LGGs
HGGs	80% of total HGGs	20% of total HGGs

### Statistical analysis

The statistical analysis was conducted using SPSS 22.0. The differences in the distribution of clinical information and tumor characteristics between the two groups are compared using chi-square and *t*-test statistical analysis. The significance level was set at 0.05. The model performance is evaluated mainly on balanced accuracy and area under the receiver operating characteristic (ROC) curve (AUC) value.

### Ethics approval

The study was conducted according to the latest version of the Declaration of Helsinki and approved by the Institutional Review Board of the Children’s Hospital of Soochow University.

### Consent to participate

Written informed consent was obtained from all participants.

## Data Availability

All data are available from the corresponding author upon reasonable request.
